# Blood-storage duration affects hematological and metabolic profiles in patients with sickle cell disease receiving transfusions

**DOI:** 10.1172/JCI192920

**Published:** 2025-07-03

**Authors:** Matthew S. Karafin, Abby L. Grier, Ross M. Fasano, Anton Ilich, David Wichlan, Ada Chang, Sonjile M. James, Hailly E. Butler, Oleg Kolupaev, Melissa C. Caughey, Daniel J. Stephenson, Julie A. Reisz, Nigel S. Key, Joshua J. Field, Jane A. Little, Steven L. Spitalnik, Angelo D’Alessandro

**Affiliations:** 1 Department of Pathology and Laboratory Medicine, University of North Carolina (UNC) at Chapel Hill, Chapel Hill, North Carolina, USA.; 2 Department of Biochemistry and Molecular Genetics, University of Colorado Anschutz Medical Campus, Aurora, Colorado, USA.; 3 Center for Transfusion Medicine and Cellular Therapies, Department of Pathology and Laboratory Medicine, Emory University School of Medicine, Atlanta, Georgia, USA.; 4 Division of Hematology and Blood Research Center, Department of Medicine, UNC at Chapel Hill, Chapel Hill, North Carolina, USA.; 5 Department of Ophthalmology, Duke University School of Medicine, Durham, North Carolina, USA.; 6 Joint Department of Biomedical Engineering, UNC and North Carolina State University, Chapel Hill, North Carolina, USA.; 7 Division of Hematology and Oncology, Department of Medicine, Medical College of Wisconsin (MCW), Milwaukee, Wisconsin, USA.; 8 Department of Pathology and Cell Biology, Columbia University Irving Medical Center, New York, New York, USA.

**Keywords:** Clinical Research, Hematology, Clinical practice, Clinical trials, Metabolomics

## Abstract

**BACKGROUND:**

Patients with sickle cell disease (SCD) frequently receive RBC units stored near the end of their permissible storage duration. We aimed to determine whether RBC storage duration influences recipient hematological, metabolic, and clinical chemistry parameters.

**METHODS:**

In a randomized, prospective, double-blind trial, 24 adults with SCD receiving chronic transfusion therapy were assigned to receive three consecutive outpatient transfusions with RBCs stored for either ≤10 days (short-stored; *n* = 13) or ≥30 days (long-stored; *n* = 11). Blood samples were collected from transfused units and from recipients at predefined time points for metabolomics, cytokine, and clinical laboratory analyses. The primary outcomes included post-transfusion hemoglobin and RBC count increments, metabolic markers of oxidative stress, iron metabolism, inflammation, and renal function.

**RESULTS:**

Transfusion of short-stored RBCs was associated with significantly higher circulating 2,3-bisphosphoglycerate levels for up to 2 weeks after transfusion. Nadir RBC counts and hemoglobin A levels were higher in recipients of short-stored RBCs. In contrast, recipients of long-stored RBCs had higher transferrin saturation and plasma iron levels, elevated markers of oxidative stress and renal dysfunction, and increased proinflammatory cytokines and immunomodulatory metabolites. Metabolomics revealed storage age–dependent alterations in glycolysis, purine, and sphingolipid metabolism. Cytokine profiles and hematologic parameters corroborated the metabolic findings, indicating improved post-transfusion metabolic and inflammatory status with short-stored RBCs.

**CONCLUSION:**

Transfusion of short-stored RBCs yielded favorable metabolic and hematologic outcomes in adults with SCD, independent of immediate clinical endpoints.

**TRIAL REGISTRATION:**

ClinicalTrials.gov NCT03704922

**FUNDING:**

National Heart, Lung, and Blood Institute (NHLBI), NIH (K23HL136787, R01HL148151, R01HL146442, and R01HL149714).

## Abstract 2

Long-stored red blood cell transfusions in sickle cell disease recipients worsen metabolic, hematological, iron metabolism and inflammation markers compared to short-stored unit transfusions.

## Graphical abstract

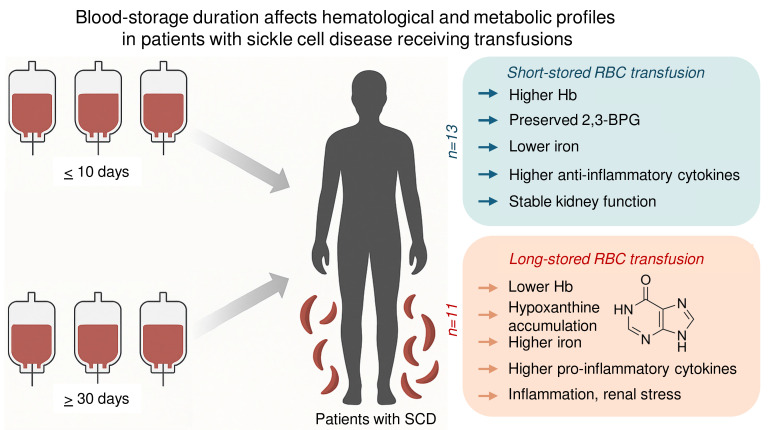

## Introduction

Over a century since its routine introduction into medical practice (1), blood transfusion remains one of the most common medical procedures, with over 11 million RBC units collected annually in the United States alone (2). RBC units can be stored in the refrigerator for up to 42 days, providing critical logistical advantages. However, extended storage induces progressive biochemical, metabolic, and morphological changes collectively termed the “storage lesion” (3). These storage-associated metabolic perturbations include slowed enzyme kinetics (4), disrupted electrolyte gradients (sodium and calcium influx, potassium efflux) (5), and diminished ATP synthesis despite supraphysiological glucose concentrations in modern storage solutions (6).

Specifically, glycolytic flux is markedly impaired (7), leading to lactate accumulation and intracellular acidification (8), further limiting glycolytic efficiency and ATP regeneration (9). Notably, critical metabolic intermediates, such as 2,3-bisphosphoglycerate (2,3-BPG) (10), rapidly decline within 2–3 weeks (11), impairing the capacity of RBCs to deliver oxygen effectively. Simultaneously, adenylate pools shift toward AMP, which is subsequently metabolized to hypoxanthine (12, 13), a biomarker closely associated with increased susceptibility to extravascular hemolysis following transfusion (3, 4, 12, 14, 15).

The reduction of 2,3-BPG not only impairs oxygen delivery but also promotes oxidative stress by increasing oxygen retention within hemoglobin molecules, facilitating Fenton and Haber-Weiss reactions in iron-rich mature RBCs. Although RBCs possess multiple antioxidant systems, persistent storage conditions overwhelm these defenses, leading to oxidative modifications of critical enzymes (16), such as GAPDH (17). These oxidative modifications redirect glucose metabolism from glycolysis to the pentose phosphate pathway (PPP) (18), affecting the availability of NADPH and reducing antioxidant defenses (19), especially glutathione (GSH) (5). ROS accumulate alongside reactive metabolites like methylglyoxal (20) and lipid peroxidation products (5), causing irreversible protein modifications (21, 22), lipid membrane alterations (23), and increased RBC rigidity (24) and fragility, ultimately leading to enhanced hemolysis. Lipid peroxidation particularly compromises membrane fluidity, facilitating splenic sequestration and extravascular clearance of RBCs (25, 26).

Despite the consistent observation of storage lesion phenomena across RBC units, considerable variability exists regarding lesion onset, progression, and severity (27), influenced by donor-specific factors including age, sex, BMI, genetic background, and environmental exposures (4, 12, 14, 15, 28, 29). These characteristics collectively determine the unit’s “metabolic age,” distinct from its chronological age (30, 31). Randomized clinical trials have generally supported standard transfusion practices, showing noninferiority compared with selective transfusion of fresher units, with caveats noted (32). Nevertheless, specific metabolic markers like kynurenine (33) and hypoxanthine (34) correlate strongly with increased RBC fragility and poorer transfusion outcomes, suggesting potential clinical relevance for tailored transfusion practices in certain patient populations.

The precise effect of RBC storage age on recipient metabolism, particularly in clinical populations, remains poorly defined. Prior omics-based investigations involving healthy volunteers (35, 36) have identified elevated levels of metabolites such as hypoxanthine and lipid peroxidation products following transfusion with older RBC units, underscoring metabolic shifts of potential clinical significance. However, important knowledge gaps persist regarding the reversibility of post-transfusion storage-induced metabolic changes (25, 37), especially concerning the PPP (36) and delayed restoration of critical metabolites like 2,3-BPG (up to 10 hours to restore 5 mM pools at a rate of 0.5 mM/h), which can measurably affect organ perfusion and oxygen kinetics (38).

Patients with sickle cell disease (SCD) present unique clinical vulnerabilities due to chronic hemolysis, systemic inflammation, endothelial dysfunction, and frequent transfusion requirements, potentially exacerbating susceptibility to adverse metabolic consequences of transfusion. Existing studies of SCD have primarily evaluated clinical endpoints such as hemoglobin increments and transfusion-related complications (39, 40), with minimal emphasis on metabolic profiling. Retrospective analyses and limited prospective studies have revealed post-transfusion metabolic disturbances (41, 42); however, these studies have often been limited by variability in patient demographics, transfusion practices (43), and incomplete characterization of the transfused RBC units.

Given the distinctive pathophysiological landscape of SCD, characterized by chronic oxidative stress and inflammation, patients may exhibit unique sensitivities to transfusion-associated metabolic disturbances. Thus, we hypothesized that transfusing RBC units stored for longer durations (long-stored, i.e., ≥30 days) would lead to discernible hematologic and metabolic alterations in recipients compared with transfusions with RBC units stored for shorter durations (short-stored, i.e., ≤10 days). These metabolic changes may occur independently of immediate clinical endpoints and reflect fundamental donor- and storage-dependent factors with potential implications for transfusion efficacy and recipient metabolic integrity.

## Results

### Short-stored and long-stored RBC units are metabolically distinct.

**Figure 1:**
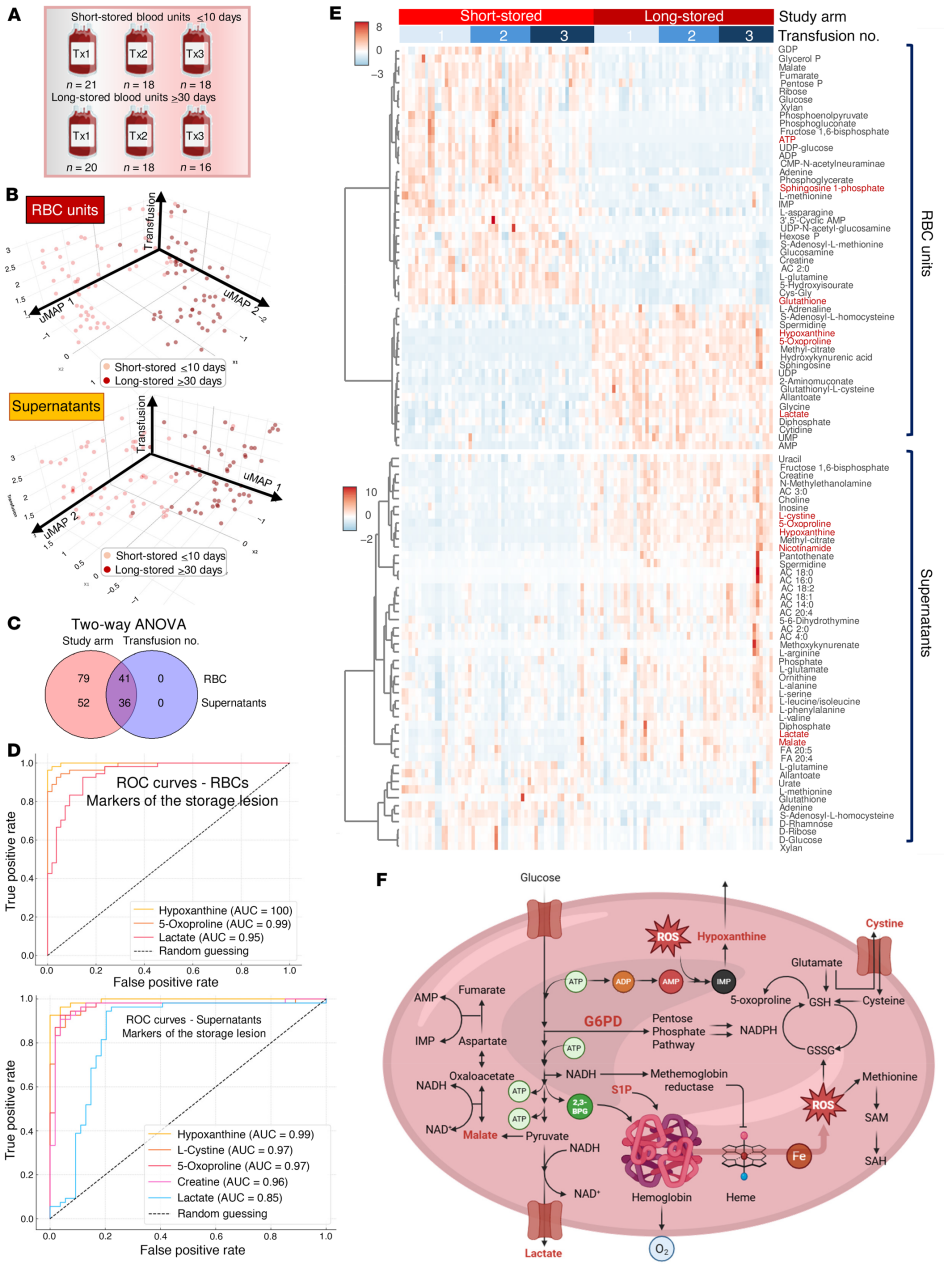
Blood units stored longer than 30 days are metabolically distinct from units stored less than 10 days. (**A**) overview of the experimental design. Numbers indicate the total units transfused per transfusion event (Tx1–Tx3). (**B**) UMAP of metabolomics data for all blood units transfused at any of the 3 transfusion events for units stored for less than 10 days (short-stored) or longer than 30 days (long-stored). (**C**) As per the study design, the age of the blood, but not the transfusion sequence, was associated with significant metabolic changes (2-way ANOVA). The transfusion event sequence is shown merely to confirm the reproducibility of storage-age–related effects across events. (**D**) ROC curves for RBC and supernatant levels of the metabolic markers of the storage lesion (14) discriminant between short-stored and long-stored units. (**E**) Heatmap of the most significant metabolic changes in RBCs and supernatants as a function of the storage age of the unit (2-way ANOVA). (**F**) Summary overview of the RBC storage metabolic lesion. Illustration was created with BioRender.com.

First, we performed metabolomics analysis on RBCs and supernatants from short-stored (≤10 days old) and long-stored (≥30 days old) RBC units, which were transfused during the 3 independent transfusion events after randomization of the recipients with SCD (Figure 1A and Supporting Data Values file; supplemental material available online with this article; https://doi.org/10.1172/JCI192920DS1). We observed significant differences, as determined by uniform manifold approximation and projection (UMAP) for both the unit’s RBCs and their corresponding supernatants (Figure 1B). These profiles showed clear separation based on storage duration, with no apparent clustering or overlap related to the type of additive solution used or the transfusion event sequence, supporting the robustness of storage age as the primary driver of the observed differences. Two-way ANOVA by RBC storage-age group and transfusion event identified a significant effect of the former, but not the latter, on the unit’s RBC and supernatant metabolomes (Figure 1C). This signature included several markers of the RBC storage lesion in the RBCs and supernatants of long-stored units, such as decreased ATP, sphingosine 1-phosphate, and GSH, and increased lactate, hypoxanthine, 5-oxoproline, cystine, and nicotinamide. Biomarker analysis (see receiver operating characteristics [ROC] curves, Figure 1D) based on these parameters confirmed high specificity and sensitivity in discriminating long-stored RBC units from short-stored ones, consistent with the literature (14). The heatmap in Figure 1E summarizes the most significant metabolic changes between the short-stored and long-stored RBCs. Figure 1F shows a summary overview of the metabolic storage lesion.

### Transfusion of short-stored RBCs affects RBC metabolism in transfusion recipients.

**Figure 2:**
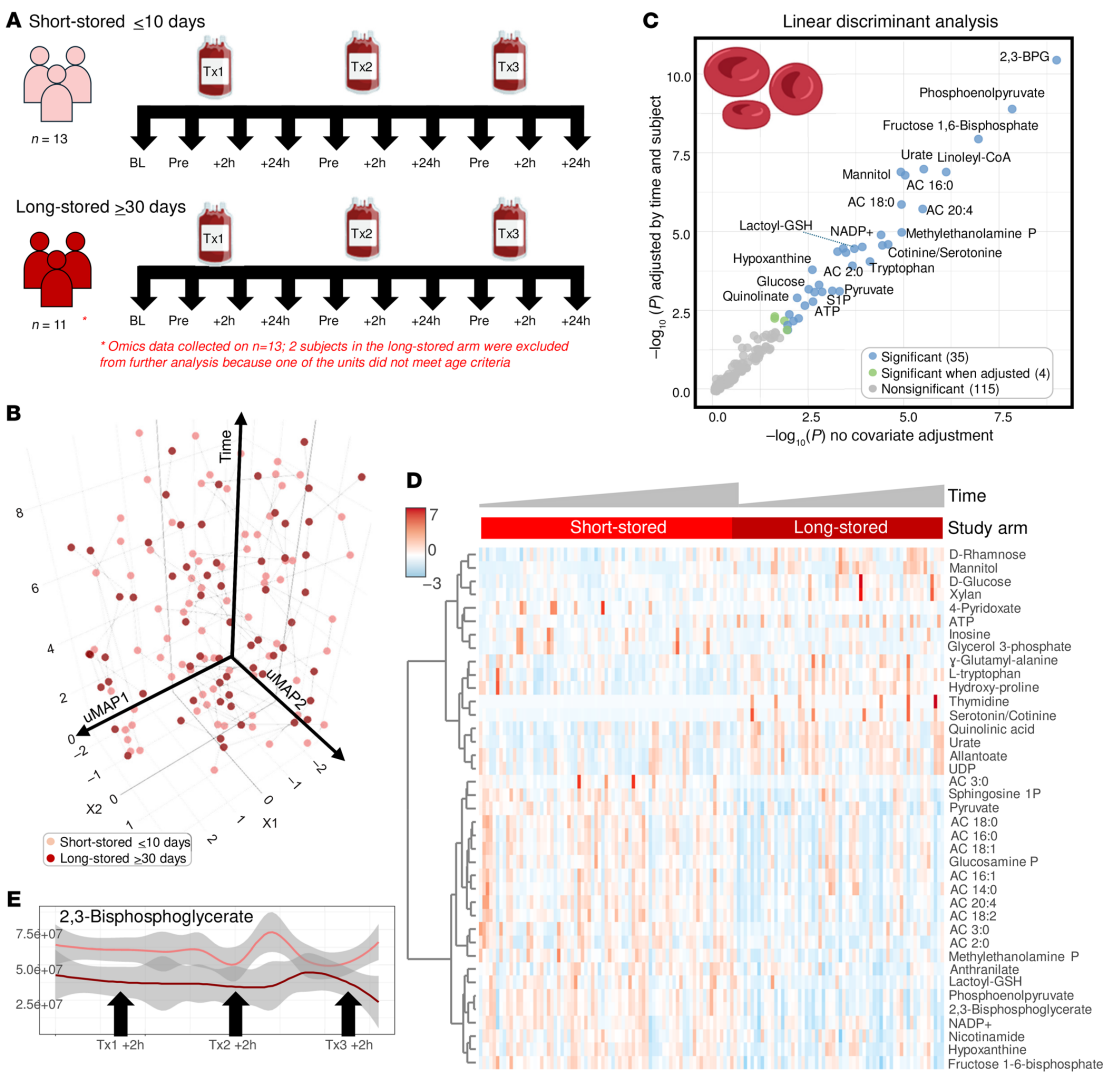
Metabolic effect of transfusion on the recipients’ RBC metabolome. (**A**) Twenty-six patients with SCD received 3 consecutive transfusions with short-stored (<10 days) or long-stored (≥30) RBCs. RBC samples were drawn for metabolomics analysis of plasma and RBCs from the recipient at baseline, before transfusion, and 2 or 24 hours after each one of the transfusion events. Two patients were excluded from the long-stored RBC study arm because some of the units they received did not match the age criteria of the study protocol. (**B**–**D**) UMAP (**B**), LDA (**C**), and heatmap (**D**) of significant RBC metabolites by time and storage age of blood by LDA. (**E**) Line plot of temporal changes in 2,3-BPG over multiple transfusions as a function of the storage age of the blood (light red and dark red for short-stored and long-stored units, respectively).

**Figure 3:**
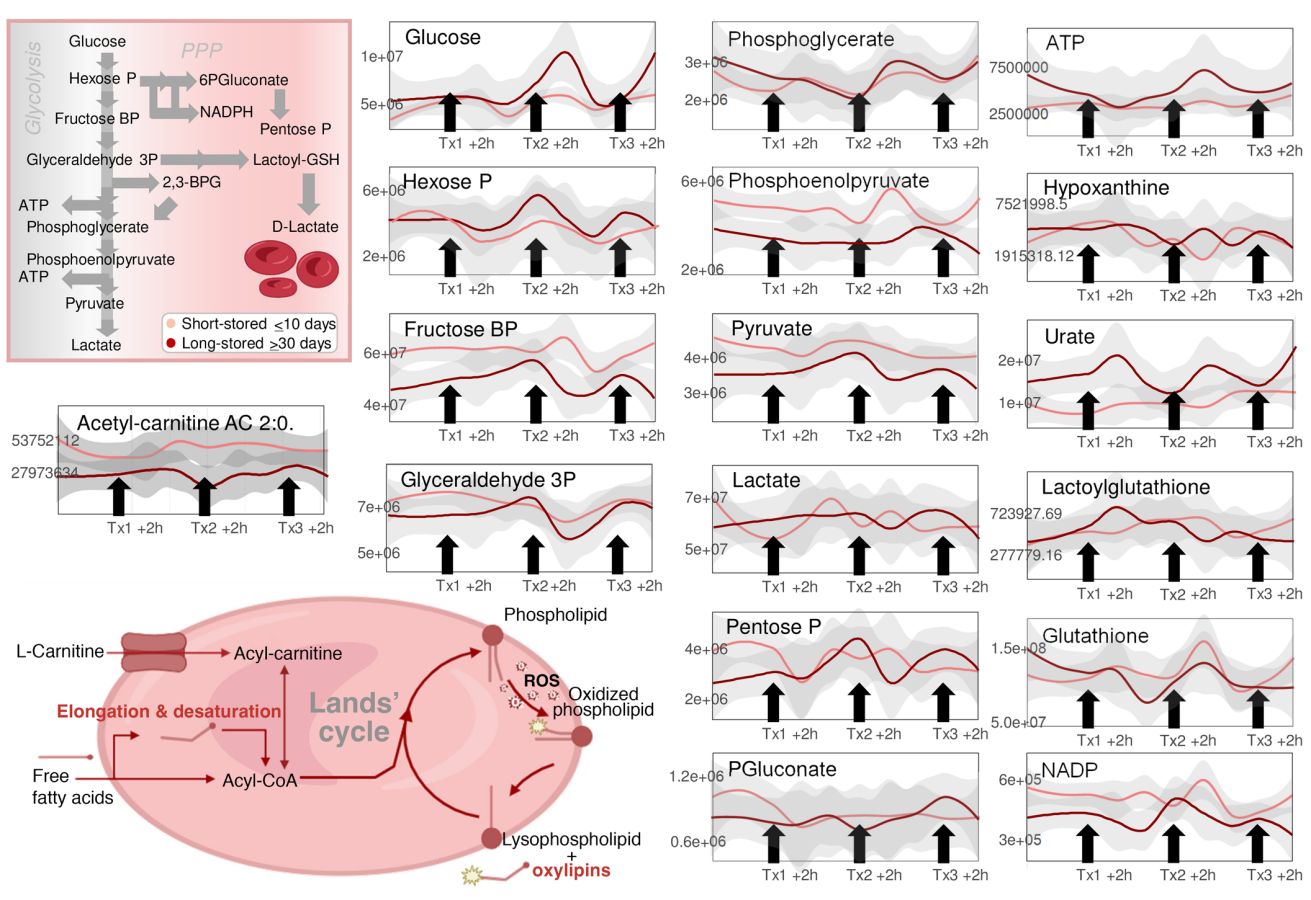
Effect of short-stored versus long-stored blood on glycolysis, the PPP, and redox homeostasis in the RBCs of transfusion recipients. Line plots show temporal changes after transfusions (Tx) 1, 2, and 3 (light red and dark red represent the median ± IQRs for short-stored and long-stored units, respectively). Vignettes were created with BioRender.com.

We performed a longitudinal assessment of the effect on recipients’ RBC metabolism of 3 independent transfusion events using either short-stored (≤10 days old) or long-stored (≥30 days old) RBCs (Figure 2A). All patients were SS genotypes and self-identified as Black, with no significant differences in age (median 28.5, 23–34) or BMI. However, the females were overrepresented (*n* = 10 of 13) in the h≥30-day study arm group compared with the ≤10-day study arm (*n* = 6 of 13) (see Supporting Data Values file). No significant differences were reported with respect to comorbidities or emergency department or hospital admission in the previous year. Most patients were transfused with 2 RBC units per event (60 of 68, 88.2%), and 80.9% of units transfused (55 of 68) were of the appropriate storage age. As such, despite metabolomics data having been collected (reported in the Supporting Data Values file), 2 study participants receiving at least 1 of the RBC units not meeting the age criteria were excluded from further analysis, leaving 13 participants in the short-stored and 11 in the long-stored RBC study arm, respectively. At baseline, 84.6% of study participants were receiving iron chelation therapy, and 15.4% were receiving hydroxyurea, with comparable distributions across the 2 study arms. Important to interpret the results described henceforth, the RBCs analyzed here were a mixture of the patients’ own RBCs and the transfused ones at an approximate ratio of 4:1. First, UMAP analysis identified stable trajectories based on the recipient rather than the randomization arm (Figure 2B). In parallel, we performed mixed-effects modeling incorporating time, study arm, their interaction, and adjustment for unit age compliance, with the results provided in the Supporting Data Values file. Consistent with the mixed-effects modeling, linear discriminant analysis (LDA) based on RBC storage age group — either unadjusted or adjusted by time and participant — identified differences in glycolysis and in purine, NAD, carnitine, and kynurenine metabolism as a function of RBC storage age (Figure 2C). An overview of the significant metabolites from this analysis is provided in Figure 2D. The levels of 2,3-BPG were higher in recipients of ≤10-day-old units through the entire study time course, with a spike noted at 2–24 hours after the second transfusion event (Figure 2E). Elevated levels of the glycolysis metabolites fructose 1,6-bisphosphate, phosphoenolpyruvate, and pyruvate, but not of glucose or hexose phosphate (combined isomers), were observed in recipients of short-stored RBCs (Figure 3). Trends in metabolites (see statistics including 2-Way ANOVA, LDA, and mixed-effects models in the Supporting Data Values file) such as RBC ATP, hypoxanthine (ATP breakdown and oxidation product), urate (oxidation product of hypoxanthine-derived xanthine), GSH, PPP intermediates (e.g., 6-phosphogluconate, pentose phosphate isomers), and lactoyl-GSH varied across the 3 transfusion time points and between study arms (Figure 3). Although some of these metabolites showed directional differences that aligned with expectations based on RBC storage lesion severity by storage age, their levels did not consistently differ between the short- and long-stored groups across all transfusion events. Therefore, we interpret these data as being indicative of complex and time-dependent metabolic remodeling,rather than as definitive evidence of a persistent storage-age effect on these individual pathways. Nicotinamide adenine dinucleotide phosphate [NAD(P)] pools were better preserved in RBCs from recipients of short-stored RBCs, with lower breakdown to nicotinamide (Figure 3), consistent with lower CD38/BST1 activation as a function of storage duration (34). Similarly, we found that acyl-carnitine pools were better preserved in recipients of short-stored RBCs, consistent with a storage-induced depletion of carnitine pools (44). Elevated mannitol was observed immediately after each transfusion event in both randomization arms, presumably due to infusion of the mannitol present in the various additive solutions (45).

### Markers of hypoxia are elevated in the plasma of recipients of long-stored RBC units.

**Figure 4:**
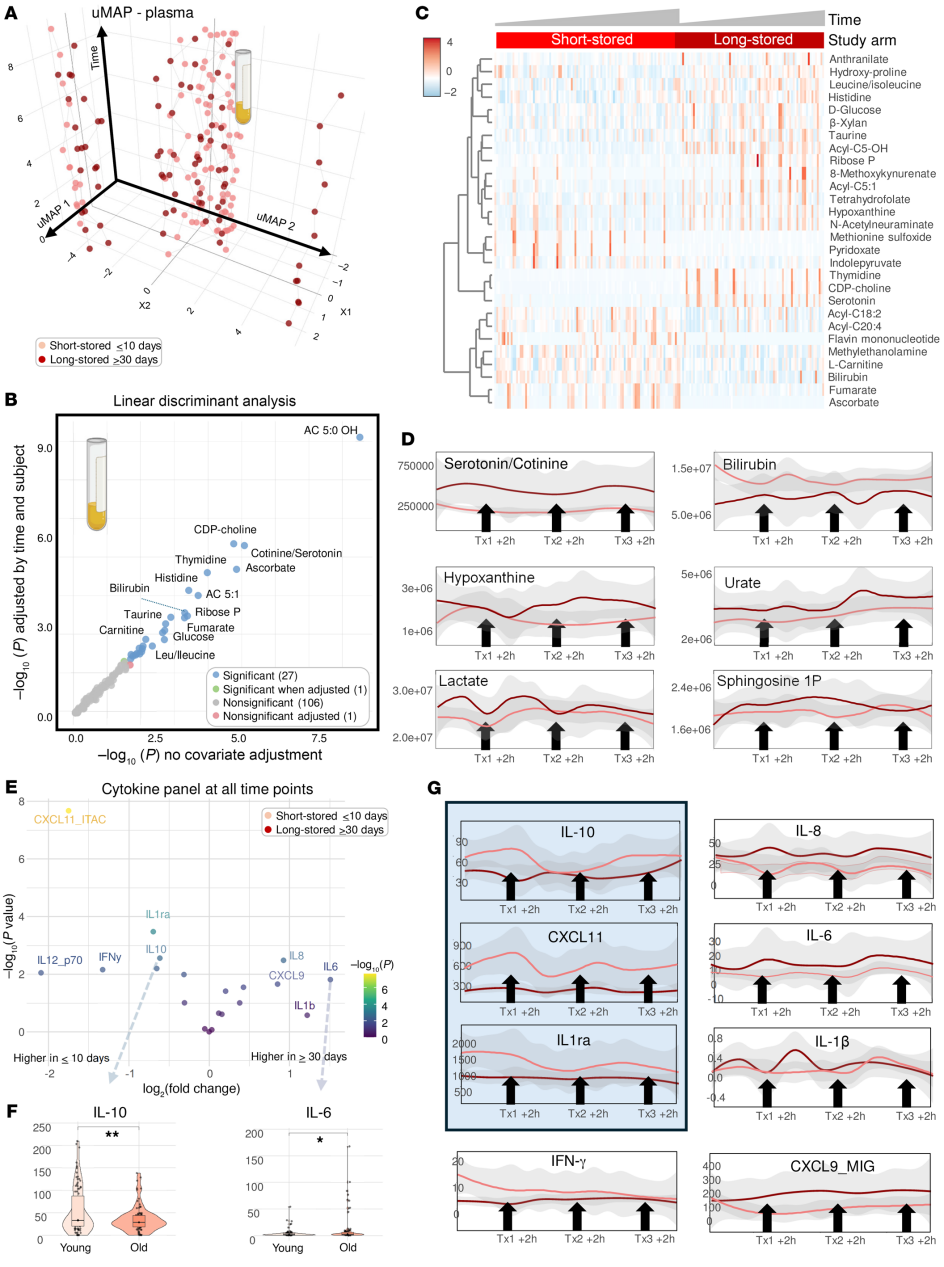
Metabolic effect of transfusion on the recipients’ plasma metabolome and cytokines. (**A**–**C**) UMAP, LDA, and heatmap of significant plasma metabolites by time and storage age of blood, as assessed by LDA. (**D**) Line plots of temporal changes in the most significantly affected plasma metabolites (as assessed by LDA) over multiple transfusions as a function of the storage age of the blood (light and dark red for short-stored and long-stored units, respectively). (**E**) Volcano plot comparing changes in circulating levels of cytokines in recipients of long- or short-stored pRBCs shows significant effects (2-tailed *t* test, adjusted) on anti- and proinflammatory cytokines (e.g., IL-10 and IL-6, respectively) at all tested time points. (**F**) Violin plots for representative antiinflammatory IL-10 and proinflammatory IL-6 being higher and lower in recipients of short-stored RBCs (Young) compared with recipients of long-stored RBCs (Old). (**G**) Line plots are shown for the time course effects for the most significantly affected pro- and antiinflammatory cytokines (2-way ANOVA). *P < 0.05, **P < 0.01.

Metabolomics analysis of plasma from recipients of short-stored versus long-stored RBC units showed more marked differences as compared with recipients’ RBCs. Except for 2 patients clustering with the long-stored RBC arm, recipients of short-stored RBCs clustered separately across UMAP 1, although their trajectories did not deviate significantly on the basis of intervention arm (Figure 4A and Supporting Data Values file). LDA identified significant effects of the age of transfused RBCs (Figure 4, B–D) on short-chain acyl-carnitines, bilirubin (unexpectedly higher in the short-stored RBC arm in the mass spectrometry [MS] analysis), ascorbate, and markers of hypoxia (e.g., hypoxanthine [ref. 46], urate [ref. 47], lactate [ref. 48], fumarate [ref. 49], and sphingosine 1-phosphate [refs. 50–52]), all of which were higher in recipients of long-stored RBCs. Recipients of long-stored RBCs also had higher baseline levels of a metabolite whose chemical and physical properties are consistent with its identification as either serotonin (platelet-derived) or cotinine (derived from smoking or other nicotine exposures) (53). An overview of all the above results is shown as a volcano plot of merged plasma and RBC data comparing the 1-hour and 24-hour post-transfusion time points versus pre-transfusion values (Supplemental Figure 1).

### Circulating levels of proinflammatory cytokines are higher in recipients of long-stored RBCs, while antiinflammatory cytokines are higher in recipients of short-stored RBCs.

Cytokine measurements identified significant increases in the levels of proinflammatory cytokines IL-6, IL-8, IL-1β, and chemokine (C-X-C motif) ligand 9 (CXCL9) in recipients of long-stored RBCs at any given time point after the first transfusion (Figure 4, E–G). On the other hand, recipients of short-stored RBCs showed higher levels of the antiinflammatory cytokines IL-12, IL-10, IL-1 receptor antagonist (IL-1ra) after transfusion (Figure 4, E–G). Proinflammatory IFN-γ levels were higher at baseline, before transfusion in patients enrolled in the short-stored RBC study arm, but its levels became comparable to those for the rest of the cohort after the first transfusion (Figure 4G).

### Transfusion of long-stored RBCs yields significantly lower RBC and hemoglobin increments and greater dysregulation of iron and renal metabolism and hemoglobin glycation.

**Figure 5:**
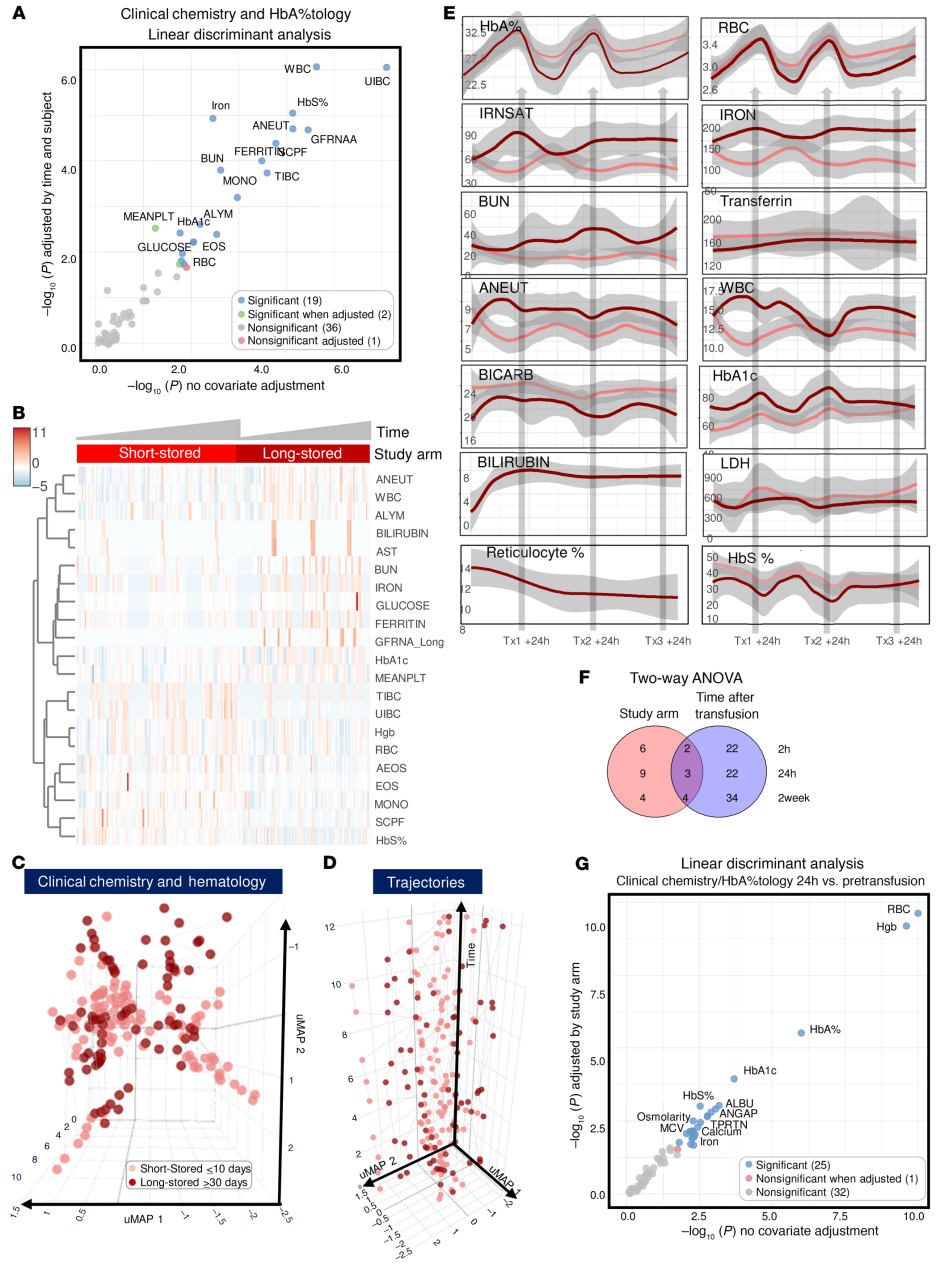
Effect of transfusion of short-stored versus long-stored blood on the recipients’ clinical chemistry panels and complete blood counts. (**A** and **B**) LDA (**A**) and heatmap (**B**) of significant clinical chemistry and hematological parameters affected by time and storage age of blood. (**C** and **D**) UMAPs (2D and 3D with temporal trajectories). (**E**) Line plots of temporal changes in the most significantly affected (2-way ANOVA in **F**) clinical chemistry and hematological parameters over multiple transfusions as a function of the storage age of the blood (light and dark red for short-stored and long-stored units, respectively). (**F**) Summary statistics of significant variables by study arm or time after transfusion and the interaction between the 2 factors. (**G**) LDA of long-stored versus short-stored blood at the 24-hour versus pre-transfusion time point for each transfusion.

Measurements of clinical chemistry and hematological parameters showed a significant effect of long-stored RBC transfusions (Figure 5, A–D, and Supporting Data Values file). These parameters were also available 2 weeks after each transfusion event, and trends for each post-transfusion time point (2 hours, 24 hours, 2 weeks), normalized to pretransfusion values, are shown in Supplemental Figure 2. Taken together, we observed cumulative effects across all 3 transfusion visits (Figure 5, B–F). (a) For RBC and hemoglobin parameters, the nadir and hemoglobin A (HbA) levels were higher in recipients of short-stored RBCs, despite higher starting percentages of HbS in patient from this arm. Transfusion of either short- or long-stored RBC units resulted in comparable drops in HbS percentages after transfusion (Figure 5, B–E); unfortunately, the reticulocyte percentages were only captured for a subset of patients from the long-stored RBC study arm at a limited number of time points (Figure 5, D and E). (b) For iron metabolism, the unsaturated iron-binding capacity was higher in recipients of short-stored RBCs, and total iron and transferrin iron saturation were higher in recipients of long-stored RBCs. Measurements of heme metabolism, in particular total and direct bilirubin, were only captured for a subset of patients in the long-stored RBC study arm, although they increased after transfusion (Figure 5E). (c) Renal function showed higher blood urea nitrogen (BUN) levels in recipients of long-stored RBCs). (d) WBC and neutrophil counts were higher for recipients of long-stored RBCs, consistent with the cytokine measurements. (e) For hemoglobin glycation, HbA1c levels were higher in recipients of long-stored RBCs. (f) For acidosis, bicarbonate levels were lower in recipients of long-stored RBCs. (g) There were comparable levels of lactate dehydrogenase (LDH), a hemolysis marker, in the 2 study arms. These effects were more marked when we directly compared trajectories 24 hours after transfusion with pre-transfusion levels (Figure 5G).

### Correlations between metabolic measurements and clinical chemistry or hematological parameters.

**Figure 6:**
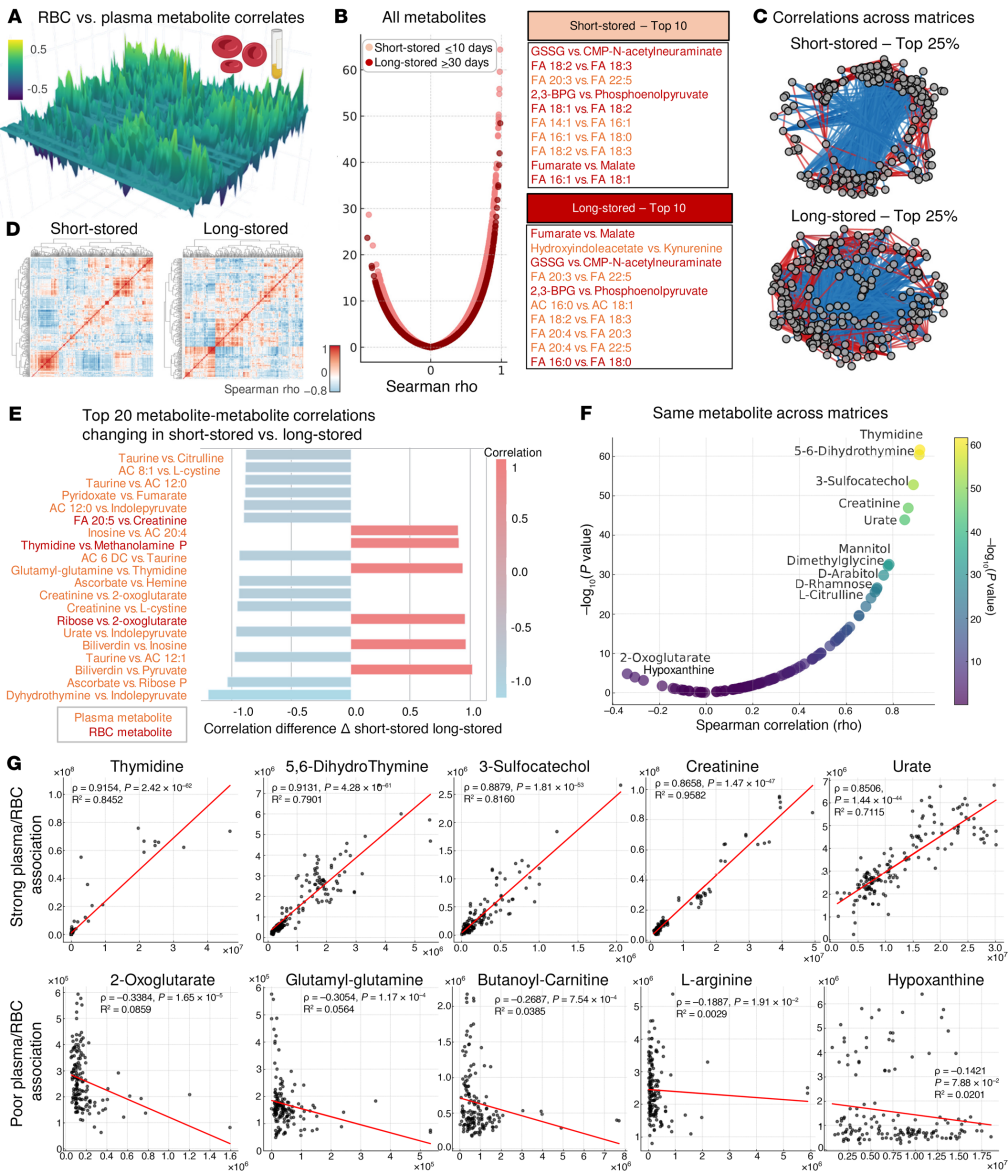
Correlation analysis of plasma versus RBC metabolic phenotypes after all transfusion events as a function of the storage age of transfused RBCs. (**A**) 3D map of Spearman’s rho correlations (*z* axis) of transfusion recipient plasma versus RBC metabolites (*x* and *y* axes). (**B**) Volcano plots of these correlations, with the top 10 most significant correlations highlighted for short-stored and long-stored blood units for plasma (light red) and RBCs (dark red). (**C** and **D**) Network and heatmap view of the correlation matrix (top 25% significant same-matrix correlations are shown). (**E**) Top 20 metabolite-metabolite correlations affected by transfusions of short-stored versus long-stored blood units. (**F** and **G**) Volcano plot (**F**) and scatter plots (**G**) of the most significantly positive and negative correlations for the same metabolite in plasma versus RBCs.

**Figure 7:**
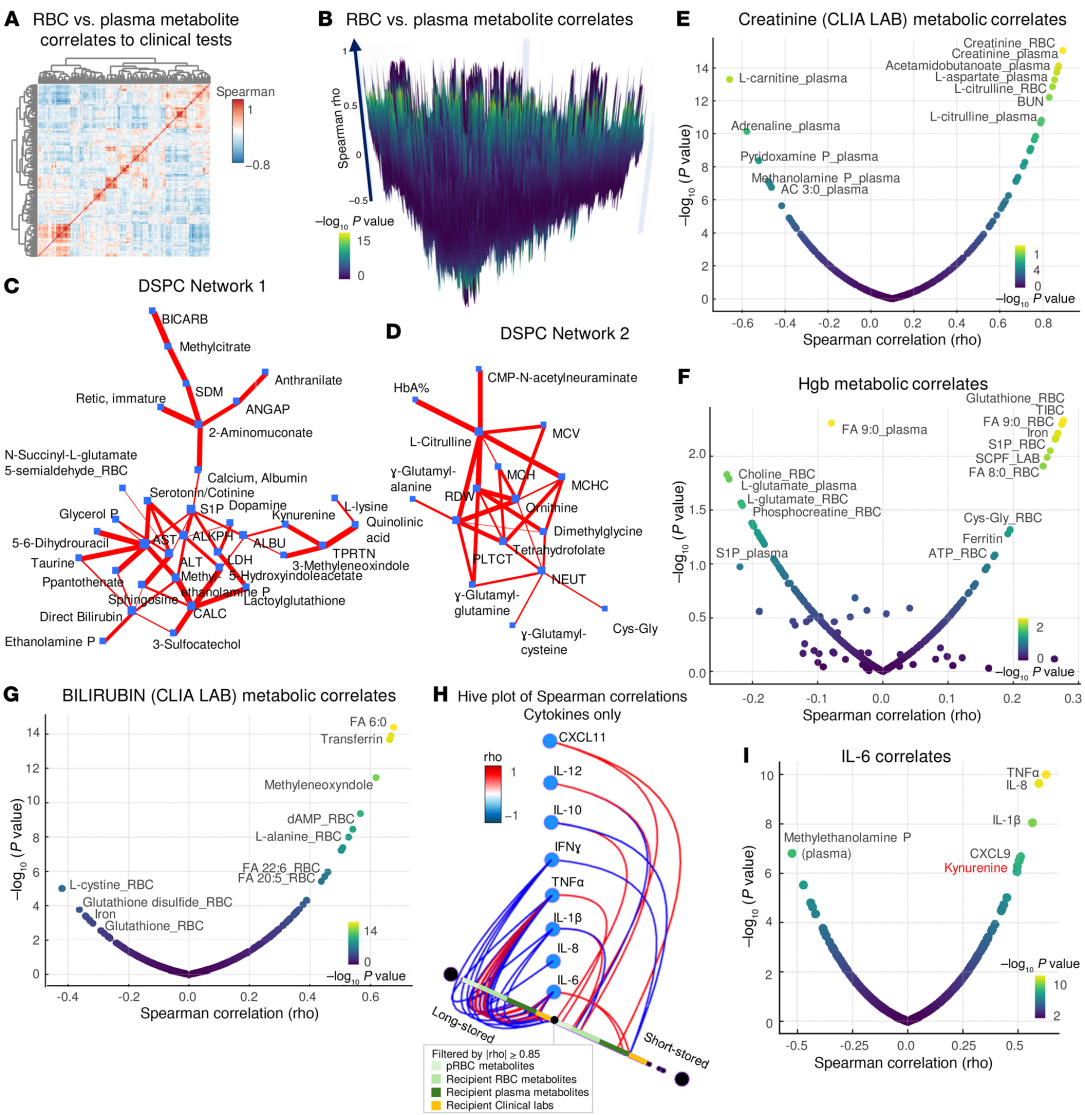
Metabolic correlates to clinical chemistry and hematological parameters. (**A**) Correlation matrix (Spearman’s rho) between metabolites and clinical chemistry and complete blood count parameters in transfusion recipients. (**B**) Same as in **A**, with the *z* axis representing Spearman’s rho positive versus negative values, and colors proportional to the –log_10_ *P* value of the correlation’s significance. (**C** and **D**) DSPC Networks 1 and 2 of the top metabolite-metabolite and metabolite-clinical covariates in this study. (**E**–**G**) Volcano plots (Spearman’s rho vs. –log_10_ *P* value for *x* and *y* axes, respectively) for clinical chemistry measurements of creatinine, hemoglobin (g/dL), and bilirubin in transfusion recipients. (**H**) Hive plot summarizing correlations (module of Spearman’s rho ≥0.85) for cytokines versus metabolites in RBC units, recipients’ RBCs, or plasma or clinical labs identifies a stronger association between proinflammatory cytokines and metabolites levels in patients with SCD who received units stored longer than 30 days. (**I**) Volcano plot of Spearman correlations to IL-6 levels in the recipient shows strong positive correlations among proinflammatory cytokines and circulating levels of kynurenine.

We then combined metabolomics measurements of recipients’ plasma and RBCs and clinical chemistry or hematological covariates to compare the main changes between the 2 study arms, irrespective of the time point (Supplemental Figure 3). We then leveraged the merged data to (a) determine the plasma versus RBC metabolites that correlated the most across matrices (Figure 6); (b) identify cross-matrix correlates (i.e., metabolites from the transfused RBCs and supernatants that correlated with metabolites in the plasma and RBCs of recipients after transfusion); and (c) identify the top omics correlates to clinical chemistry or hematological parameters (Figure 7).

The first analysis identified a cluster of strongly correlated intra-matrix metabolites (Figure 6A), especially with respect to fatty acid (FA) metabolism (e.g., FA 18:2 vs. FA 18:3 in RBCs), glycolysis (e.g., 2,3-BPG vs. phosphoenolpyruvate), and carboxylic acid metabolism (e.g., fumarate vs. malate); most of these overlapped between recipients of short-stored or long-stored RBCs (Figure 6B). A network overview of the top 25% of correlations (by Spearman’s rho and *P* value) is shown in Figure 6C, with the respective matrix view in the heatmap in Figure 6D. After noting a robust core of intra-matrix metabolite-metabolite correlates, we then sought to understand which of these correlations were most significantly altered by the study arm, an analysis suggestive of an effect of the selective transfusion of short- or long-stored RBCs on that specific metabolic reaction subnetwork (Figure 6E). Deltas of short- or long-stored RBC correlations identified altered taurine, citrulline/creatinine, cystine/ascorbate, bilirubin, pyruvate, thymidine, FA, and carnitine metabolism as the most affected pathways as a function of the storage age of the transfused RBC units (Figure 6E).

We then sought to determine the metabolites whose levels correlated the most or the least between matched plasma and RBCs from the same transfusion recipient (Figure 6F), identifying a strong cross-matrix reproducibility in the levels of thymidine and 5,6-dihydrothymine, sulfocatechol, creatinine, and urate (Figure 6G). In contrast, poor correlations were observed between plasma and RBC levels of hypoxanthine, l-arginine, 2-oxoglutarate, glutamyl-glutamine, and butanoyl-carnitine, suggesting matrix-specific metabolism (e.g., catabolism of hypoxanthine to urate (Supplemental Figure 4).

Correlation of metabolomics data to clinical chemistry or hematological parameters (Figure 7, A and B) identified several key clusters. The top 2 clusters are highlighted in the Debiased Sparse Partial Correlation (DSPC) network in Figure 7, C and D, and identified a strong association between (a) network 1 (Figure 7C): acidosis and circulating carboxylates (e.g., methylcitrate), transaminases (aspartate transferase [AST], alanine transaminase [ALT]), sphingosine 1-phosphate/taurine, direct bilirubin (clinical chemistry), ethanolamine/sphingolipid metabolism, total protein/albumin, and kynurenine metabolism (kynurenine, quinolinic acid); and (b) network 2 (Figure 7D): HgA and citrulline, MCH, MCV, MCHC, RDW, ornithine, and neutrophil and GSH metabolism (γ-glutamyl-cysteine/glutamine; cysteinyl-glycine).

Focusing on selected clinical chemistry covariates, such as creatinine (Figure 7E) confirmed the quality of the MS-based metabolomics data, with a strong association between Clinical Laboratory Improvement Amendments–regulated (CLIA-regulated) clinical chemistry assays for creatinine and the MS measurements in these patients (Supplemental Figure 5). Of note, several metabolites involved in arginine metabolism (e.g., citrulline and aspartate) also correlated with creatinine levels.

Hemoglobin levels strongly and positively correlated with total GSH, iron levels, sphingosine 1-phosphate (in RBCs), and octanoic or nonanoic acid (FA 8:0 and 9:0) (Figure 7F). Clinical chemistry measurements of bilirubin, for the subset of recipients of long-stored RBC transfusions for which this measurement was available, were negatively associated with total GSH pools (both reduced and oxidized GSH) (Figure 7G).

Finally, analysis of a correlation of metabolites from RBC units (either the RBCs or supernatants) with circulating plasma or RBC metabolites in the recipients did not identify specific metabolites whose levels in the RBC unit affected post-transfusion levels of the same metabolite in the recipient (e.g., no association between hypoxanthine in stored RBCs or supernatants and its levels in post-transfusion plasma or RBCs; Supplemental Figure 6). Even when significant associations were noted (e.g., sphingosine 1-phosphate in stored RBCs and circulating RBCs in transfusion recipients; Supplemental Figure 7), the actual correlation was not compelling. However, we noted specific metabolite (RBC unit) to metabolite (recipient) associations, including a negative association between hypoxanthine in the RBC units and urate in transfusion recipients.

On the other hand, metabolite levels, in both transfused RBCs and in the plasma and RBCs of transfusion recipients strongly correlated (module of Spearman rho ≥0.85) with several proinflammatory cytokines in recipients of long-stored RBCs and with antiinflammatory cytokines in recipients of short-stored RBCS (Figure 7H). A network of strongly and positively intercorrelated proinflammatory cytokines (above all, IL-6, TNF-α, IL-8) (Figure 7I) was linked to circulating levels of kynurenine.

## Discussion

The present study provides what to our knowledge is the first direct evidence of a metabolic effect of the storage age of transfused RBCs on clinical chemistry or hematological parameters and related metabolic measurements in transfusion recipients in a prospective randomized trial involving adults with SCD. The effect of the storage lesion is well established and confirmed here by direct measurements of the metabolic changes in supernatant and RBCs of short- and long-stored RBC units. These results confirm a depletion of adenylate (34) and NAD(P) and GSH pools (54); the accumulation of byproducts of their oxidation and catabolism (e.g., hypoxanthine [ref. 34], nicotinamide [ref. 34], and 5-oxoproline [ref. 14]); and depletion of acyl-carnitine pools (44), methionine (55), and sphingosine 1-phosphate (50).

Nonetheless, questions remained whether these metabolites affected the bloodstream characteristics of patients requiring allogeneic transfusions, such as the individuals with SCD enrolled in the present study. The current results demonstrate that both recipients’ plasma and RBCs were significantly affected, with one notable effect being related to increased circulating levels of mannitol (a storage additive component), irrespective of the storage age of the RBC unit. More physiologically relevant, transfusion of short-stored RBC units was associated with significantly higher circulating levels of 2,3-BPG in recipients; this result is consistent with improved oxygen kinetics in recipients of short-stored RBCs and supports recent studies of renal perfusion (38). While transfusion of short-stored RBC units was associated with higher recipient levels of glycolysis intermediates such as 2,3-BPG and phosphoenolpyruvate, interpretation of downstream metabolites such as hypoxanthine, 6-phosphogluconate, and lactoyl-GSH requires caution. These metabolites exhibited variability across the transfusion timeline and did not consistently segregate by study arm. As such, although trends in hypoxanthine and its catabolite urate aligned with known oxidative pathways (12, 34, 47), these findings likely reflect a combination of storage lesion severity, recipient-specific factors, and the temporal dynamics of post-transfusion metabolic remodeling rather than a uniform storage-age effect. These metabolomics results were corroborated by clinical chemistry measurements of acidosis, with significantly lower bicarbonate detected in the plasma of recipients of long-stored RBCs. Lower sphingosine 1-phosphate in recipients with SCD is clinically relevant because this metabolite stabilizes deoxyhemoglobin, thereby potentially promoting crystallization of sickle hemoglobin in these patients (39, 40, 51). Similarly, higher hypoxanthine levels in long-stored RBCs and their supernatants positively correlated with plasma and RBC urate levels in transfusion recipients, suggesting rapid metabolism of hypoxanthine to urate by xanthine dehydrogenase/oxidase, a reaction that also generates hydrogen peroxide. In this context, the negative association between hypoxanthine levels and the post-transfusion circulatory capacity of stored RBCs may not be just correlative, but may also be mechanistically explained by the utilization of this metabolite as a substrate for pro-oxidant reactions following transfusion, consistent with mechanistic models of reperfusion injury upon ischemic hypoxia (49).

Transfusion of long-stored RBCs was associated with poorer overall efficacy (e.g., lower HgA and RBC retention over the 3 transfusion events) and increased circulating iron levels and transferrin saturation, all consistent with elevated hemolysis and prior results in healthy recipients of autologous RBCs stored for more than 35 days (56). This observation is explained, in part, by better preservation of antioxidant systems (e.g., GSH pools, PPP metabolites) in RBCs of recipients of short-stored RBC units in the current study. Transfusion of long-stored RBCs was associated with renal dysfunction (increased BUN and creatinine, the latter observed by both clinical chemistry and MS-based assays); this is likely due to increased hemolysis and membrane lipid remodeling in long-stored RBCs (57) resulting from depletion of carnitine pools (44). In addition, the association between these parameters and kynurenine and its metabolites (e.g., quinolinic acid, a neurotoxicant) is consistent with the recently reported linkage of dysregulated kynurenine metabolism in RBCs at the time of donation with increased osmotic fragility and lower post-transfusion hemoglobin increments (33) or elevated proinflammatory cytokines like IL-6 and IFN-γ in the context of COVID-19 (58) or after strenuous exercise (59). In the present study, circulating kynurenine levels were here linked to IL-6 and to a series of proinflammatory cytokines. Elevated WBC and neutrophil counts in recipients of long-stored RBCs is consistent with activation of proinflammatory processes in transfusion recipients and with the established roles of metabolites — e.g., lactate (60), citrulline (61), sphingosine 1-phosphate (62–64), and carboxylic acids — e.g., fumarate and succinate (65) in immunometabolic reprogramming toward proinflammatory phenotypes. These observations were corroborated by direct measurements of circulating cytokines, which identified elevation of the proinflammatory cytokines and key drivers of poor outcomes in patients with sickle cell anemia (66) — IL-6, IL-1β, IL-8, and CXCL9 — in recipients of long-stored RBCs, whereas recipients of short-stored RBCs had higher levels of the antiinflammatory cytokines IL-10, IL-12, and CXCL11. Of note, CXCL11 can drive the polarization of CD4^+^ T cells into IL-10–producing Tr1 cells (67), which are a type of Treg known for their immunosuppressive properties. As such, CXCL11 has been linked to the modulation of tolerogenic immune responses, which is important in light of the clinical role of alloimmunization in patients with SCD undergoing chronic transfusions (68). On the other hand, proinflammatory CXCL9 and CXCL10 were elevated after transfusion of long-stored, but not short-stored, RBCs. Transfusion of short-stored RBCs also lowered proinflammatory IFN-γ levels, which were higher before transfusion in the patients enrolled in that study arm. Notably, the metabolism of arginine to ornithine was recently linked to the age of the donor, the storage age of the donated RBCs, and, ultimately, to post-transfusion hemoglobin increments (29). Finally, altered post-transfusion levels of glucose and HbA1c in recipients of long-stored RBC units is consistent with previous hypotheses (69) and is reported here for the first time to our knowledge in a prospective, randomized clinical trial.

In addition to the hematologic and metabolic outcomes, we systematically captured severe and nonsevere adverse events, including vaso-occlusive episodes (VOEs) and hospitalizations. While the present study was primarily powered to detect metabolic differences, exploratory analyses revealed that individuals randomized to receive longer-stored RBC units (≥30 days) had a numerically higher incidence of pain crises and nonsevere adverse events requiring medical attention. Although these observations suggest that storage duration may influence clinical outcomes in patients with SCD, the study was not powered to definitively evaluate these endpoints. A comprehensive analysis of adverse event rates, hospitalization frequency, and clinical predictors of post-transfusion morbidity in this cohort will be reported separately in a forthcoming companion manuscript. These preliminary findings, however, further highlight the potential importance of donor and storage factors in optimizing transfusion support for individuals with SCD.

This study has several notable limitations. First, the small sample size (13 participants per arm was originally planned, with 11–13 participants ultimately analyzed) limits the statistical power for robust detection of subtle differences or conclusive establishment of causation between RBC storage age and clinical outcomes. Recruitment targets were affected primarily by changes in clinical practice favoring exchange transfusions and interruptions due to the COVID-19 pandemic. Second, despite using rigorous statistical modeling (e.g., repeated measures and mixed-effects modeling) to control for donor and unit variability (e.g., hemoglobin dosage, processing sites, anticoagulants), residual confounding likely persists. Third, the study design did not include a crossover arm, which would have helped control for individual patient variability, thus potentially affecting the interpretation of some metabolic and hematological parameters. Fourth, alloimmunization status and comprehensive antigen-matching details beyond cEK were not systematically captured, limiting the ability to explore their effect on transfusion efficacy and RBC survival; these represent important areas for future studies. Fifth, while cytokine measurements and WBC flow cytometric data were obtained, these complex analyses were beyond the scope of the current manuscript and will be addressed comprehensively in a companion manuscript currently under preparation. Sixth, although hydroxyurea and iron chelation therapies were recorded and distributed similarly between groups, the potential effects of these disease-modifying treatments on transfusion outcomes cannot be excluded. Last, most participants (84.6%) underwent iron chelation, and a smaller subset (15.4%) received hydroxyurea; variations in these therapies might influence transfusion outcomes and are explicitly detailed in the demographics data (see Supporting Data Values file). Further large-scale studies are required to validate these findings and their broader clinical implications, particularly in the context of current clinical practices.

In conclusion, this prospective randomized clinical trial identified clinical chemistry and hematological and metabolic effects resulting from the storage age of RBC units by longitudinally monitoring of 3 independent transfusion events in patients with SCD undergoing chronic transfusion. Our results indicate that selective transfusion of RBC units stored for ≤10 days had some beneficial effects on metabolic regulators of oxygen kinetics, whereas transfusion of long-stored RBCs was associated with potentially harmful increased circulating markers of hypoxia, hemolysis, iron metabolism, inflammation, and renal dysfunction.

## Methods

### Sex as a biological variable.

The study enrolled 10 female and 3 male patients in the ≥30-day-old RBC units arm and 6 female and 7 male patients in the ≤10-day-old RBC units arm.

### Study design.

This study is part of a prospective, randomized, blinded pilot clinical trial conducted at 3 sites (MCW, UNC, and Emory University) under protocols approved by their respective IRBs. Between 2017 and 2024, we recruited 26 adults (age range, 16–60 years) with SCD who were treated with chronic outpatient RBC transfusions (i.e., 1 or 2 RBC units transfused every 3–8 weeks per a medically defined protocol) (70). Study participants who provided written informed consent were randomized to receive ≥30-day-old (*n* = 13) or ≤10-day-old (*n* = 13) units for 3 consecutive outpatient simple transfusion events. Three participants receiving units that did not meet the (RBC unit) age criteria were excluded from the ≥30-day-old group, despite the fact that the samples were collected and metabolomics analyses were performed (see Supporting Data Values file). A baseline venous blood sample for flow cytometry was obtained just prior to the first randomized transfusion.

The study participants were asked to hold off their iron chelation for 72 hours before each study transfusion so that the post-transfusion change in circulating serum iron could be clearly defined. Pretransfusion unit link samples were obtained from each RBC unit for flow cytometric and metabolomics analyses. RBC and supernatant samples (100 μL via a 10-minute centrifugation at 4°C at 1,500*g*) were collected from residual transfusates from each unit immediately after transfusion. Patients’ venous blood samples were also obtained before transfusion and 2 and 24 hours after transfusion for flow cytometric and metabolomics analyses (100 μL via a 10-minute centrifugation at 4°C at 1,500*g*). Participants completed standardized diaries each day until the end of the study to document subjective symptoms of infection, pain, and emergency department or hospital utilization. All data were captured with standardized case report forms and entered into an electronic database. This database also included a record of basic medical and surgical histories (e.g., age, sex, reason for receiving chronic transfusion therapy, past medical history, medication list, previous hospitalizations) (70).

For cytokine measurements, all Luminex assays were performed by the UNC Respiratory TRACTS Core Laboratory according to the manufacturer’s instructions, with the exception of the standard curve, which was extended to a 7-point curve for all assays to increase the quantifiable range. All Luminex assays were read on an Intelliflex Single Reporter system using the high-sensitivity settings.

Consecutive outpatient transfusions involved age-appropriate, crossmatch-compatible, CEK antigen–matched, ABO/RhD-compatible RBC units negative for the sickle trait, and sickle-negative RBC units stored in either additive solution 1 (AS-1), AS-3, or AS-5. Detailed patient demographics (all SS genotypes), including age, sex, and baseline laboratory data, are provided in the Supporting Data Values file. Information on chronic therapies, including hydroxyurea use and iron chelation therapy, was collected at enrollment and also summarized in the Supporting Data Values file. Neither the patient nor the clinical staff overseeing the patient’s participation in the trial, including the study’s principal investigator and study coordinators, had access to the treatment arm assignment. However, no alterations were made to the labels on the RBC units; therefore, unintended unblinding of study participants was possible. Medical infusion clinic personnel physically providing the RBC transfusions verified product and patient identity according to hospital-specific procedures.

### Sample preparation for metabolomics.

Metabolites from plasma and supernatants from RBC units were extracted at a ratio of 1:25 and from RBCs at a ratio of 1:10 with cold MeOH/MeCN/H_2_O (5:3:2, v/v/v) and 100% MeOH for metabolomics/oxylipins/bile acid analysis and lipidomics analysis, respectively. Suspensions were vortexed vigorously for 30 minutes at 4°C. Insoluble material was pelleted by centrifugation (18,213*g*, 10 minutes, 4°C), and supernatants were isolated for analysis by ultra-high-pressure liquid chromatography–MS (UHPLC-MS) (71).

### UHPLC-MS analysis.

A Vanquish UHPLC system (Thermo Fisher Scientific) was coupled to an Orbitrap Exploris 120 mass spectrometer (Thermo Fisher Scientific). Sample injections (10 μL) were resolved across a 2.1 × 150 mm, 1.7 μm Kinetex SB-C18 column (Phenomenex) using a 5-minute, reversed-phase gradient, as described previously (71) (see details in Supplemental Methods). The run order of samples was randomized, and technical replicates were included to assess quality control. Raw files were converted to .mzXML using RawConverter. The resultant files were processed with El-Maven (Elucidata) alongside the Kyoto Encyclopedia of Genes and Genomes (KEGG) database for metabolite assignment and peak integration as previously described (34, 72).

### Statistics.

Raw metabolomics data were autoscaled using MetaboAnalyst 5.0 (73). Two-way ANOVA, repeated-measures ANOVA, or a 2-tailed Student’s *t* test was used to determine significance by matrix, treatment arm over time, or at a specific time point. A *P* value of less than 0.05 (FDR adjusted *P* < 0.05 for ANOVA) was considered significant. UMAP, a nonlinear dimensionality reduction technique that transforms high-dimensional data into a lower-dimensional representation while preserving its structure, was used to analyze metabolomics data as a function of study arm and time (74). To account for repeated measures within individuals and to minimize the effect of confounding variables such as donor hemoglobin dosage, donor biology, processing site differences, and additive solution type, we performed additional analyses using mixed-effects models with random intercepts for study participants and fixed-effects for storage duration, transfusion event, and additive solution (see details in Supplemental Methods). This approach allowed for robust adjustment for both intra-subject and inter-unit variability. In-house code for R-4.4.1 and MetaboAnalyst were used to perform nonparametric, 2-tailed Spearman’s rank correlation tests with the non-normalized, raw data to generate correlation plots for variables of interest, including correlations across matrices (RBCs and supernatants from transfused units, plasma, and RBCs in transfusion recipients) for metabolomics data and/or clinical biochemistry or hematological variables. This software was also used to calculate and plot linear regressions and to generate a central network plot. Additional figures were generated using GraphPad Prism 10 (GraphPad Software) and BioRender.com (see figure legends for BioRender credits).

### Data availability.

All raw data and elaborations are available in Supporting Data Values file.

## Author contributions

MSK, JJF, and JAL designed the clinical trial and reviewed the manuscript. MSK, ASG, RMF, AI, DW, AC, SMJ, HEB, OK, MCC, DS, JAR, and NSK implemented the study and reviewed the manuscript. AD, MSK, and SLS helped write the manuscript. AD performed analysis.

## Supplementary Material

Supplemental data

ICMJE disclosure forms

Supporting data values

## Version 1 07/03/2025

In-Press Preview

## Version 2 09/02/2025

Electronic publication
