# Supplementary material for: Blood-storage duration affects hematological and metabolic profiles in patients with sickle cell disease receiving transfusions
Source: J Clin Invest. 2025 Jul 3;135(17):e192920. doi: 10.1172/JCI192920 (PMC12404753; doi:10.1172/JCI192920)
Supplement: Supplemental data [file jci-135-192920-s320.pdf]

SUPPLEMENTARY MATERIAL for

Blood-storage duration affects hematological and metabolic profiles in patients with sickle cell disease receiving transfusions

Matthew S. Karafin,[1] Abby S. Grier,[2] Ross M. Fasano,[3] Anton Ilich,[4] David Wichlan,[4] Ada Chang,[4] Sonjile M. James,[3] Hailly E. Butler,[3] Oleg Kolupaev,[5] Melissa C. Caughey,[6] Daniel Stephenson,[2] Julie A. Reisz,[2] Nigel S. Key,[4] Joshua J. Field,[7] Jane A. Little,[4] Steven L. Spitanik,[8] Angelo D'Alessandro[2,*]

1) Department of Pathology and Laboratory Medicine, University of North Carolina, Chapel Hill, NC, United States;
2) Department of Biochemistry and Molecular Genetics, University of Colorado Anschutz Medical Campus, Aurora, CO, USA;
3) Center for Transfusion Medicine and Cellular Therapies, Department of Pathology and Laboratory Medicine, Emory University School of Medicine, Atlanta, GA.
4) Division of Hematology and Blood Research Center, Department of Medicine, University of North Carolina at Chapel Hill, Chapel Hill, NC.
5) Department of Ophthalmology, Duke University School of Medicine, Durham, NC.
6) Joint Department of Biomedical Engineering, University of North Carolina and North Carolina State University, Chapel Hill, NC.
7) Division of Hematology and Oncology, Department of Medicine, Medical College of Wisconsin, Milwaukee, WI.
8) Department of Pathology and Cell Biology, Columbia University Irving Medical Center, New York City, New York, USA.

***Corresponding authors:**
Matthew S Karafin, MD
Department of Pathology and Laboratory Medicine
University of North Carolina, Chapel Hill, NC, United States.
E-mail: Matthew.Karafin@unchealth.unc.edu

Angelo D'Alessandro, PhD
Department of Biochemistry and Molecular Genetics
University of Colorado Anschutz Medical Campus
12801 East 17th Ave., Aurora, CO 80045
Phone # 303-724-0096
E-mail: angelo.dalessandro@cuanschutz.edu
www.dalessandrolab.com

TABLE OF CONTENTS

SUPPLEMENTARY METHODS 2
SUPPLEMENTARY REFERENCES 2
SUPPLEMENTARY FIGURES 2
SUPPLEMENTARY FIGURE 1 3
SUPPLEMENTARY FIGURE 2 4
SUPPLEMENTARY FIGURE 3 5
SUPPLEMENTARY FIGURE 4 6
SUPPLEMENTARY FIGURE 5 7
SUPPLEMENTARY FIGURE 6 8
SUPPLEMENTARY FIGURE 7 9

SUPPLEMENTARY TABLE 1 .... XLS

SUPPLEMENTARY METHODS

Detailed metabolomics methods

Filtered extracts were transferred to an ultra-high-pressure liquid chromatography (UHPLC-MS — Vanquish) equipped with a plate charger. A blank containing a mix of standards detailed before (1) and a quality control sample (the same across all plates) were injected 2 or 5 times each per plate, respectively, and used to monitor instrument performance throughout the analysis. Metabolites were resolved on a Phenomenex Kinetex C18 column (2.1 x 30 mm, 1.7 um) at 45 °C using a 5-minute gradient method in positive and negative ion modes (separate runs) over the scan range 65-975 m/z(2) exactly as previously described (3). The UHPLC was coupled online to a Q Exactive mass spectrometer (Thermo Fisher). The Q Exactive MS was operated in negative ion mode, scanning in Full MS mode (2 µscans) from 90 to 900 m/z at 70,000 resolution, with 4 kV spray voltage, 45 sheath gas, 15 auxiliary gas. Following data acquisition, .raw files were converted to .mzXML using RawConverter then metabolites assigned and peaks integrated using ElMaven (Elucidata) in conjunction with an in-house standard library (4).

Statistical analysis of metabolomics data

To evaluate the impact of storage duration on recipient RBC and plasma metabolomes, we applied mixed-effects linear modeling to each metabolite individually. Only metabolites with at least 10 non-missing observations across subjects and timepoints were included, ensuring sufficient degrees of freedom for model stability.
For each metabolite, the following model was fit:

$$Y_{ij}=\beta_0+\beta_1(\mathrm{Time}_{ij})+\beta_2(\mathrm{StudyArm}_{ij})+\beta_3(\mathrm{Time}\times\mathrm{StudyArm}_{ij})+\beta_4(\mathrm{AgeCriteria}_{ij})+ u_i+\epsilon_{ij}$$

where:

- Y_{ij} is the metabolite concentration for subject i at timepoint j,
- **Time** represents the transfusion event timepoint,
- **StudyArm** denotes randomization to <10-day vs >30-day stored RBC units,
- **Time × StudyArm** captures the interaction between transfusion timing and storage age,
- **AgeCriteria** is a binary covariate adjusting for whether all transfused units met the intended storage duration (1 = yes, 2 = no),
- u_i is a random intercept accounting for repeated measures within subjects,
- ϵ_{ij} is the residual error.

Models were fitted using the statsmodels package (v0.14.0) in Python, using the mixedlm function with maximum likelihood estimation.
P-values were extracted for fixed effects (Time, Study Arm, Time × Arm interaction, and Age Criteria adjustment). Results were reported only for metabolites meeting data completeness thresholds to ensure model validity.

SUPPLEMENTARY REFERENCES

1. Stefanoni D, Shin HKH, Baek JH, Champagne DP, Nemkov T, Thomas T, et al. Red blood cell metabolism in Rhesus macaques and humans: comparative biology of blood storage. *Haematologica.* 2020;105(8):2174-86.
2. Nemkov T, Yoshida T, Nikulina M, and D'Alessandro A. High-Throughput Metabolomics Platform for the Rapid Data-Driven Development of Novel Additive Solutions for Blood Storage. *Front Physiol.* 2022;13:833242.
3. Nemkov T, Reisz JA, Gehrke S, Hansen KC, and D'Alessandro A. High-Throughput Metabolomics: Isocratic and Gradient Mass Spectrometry-Based Methods. *Methods Mol Biol.* 2019;1978:13-26.
4. Nemkov T, Hansen KC, and D'Alessandro A. A three-minute method for high-throughput quantitative metabolomics and quantitative tracing experiments of central carbon and nitrogen pathways. *Rapid communications in mass spectrometry : RCM.* 2017;31(8):663-73.

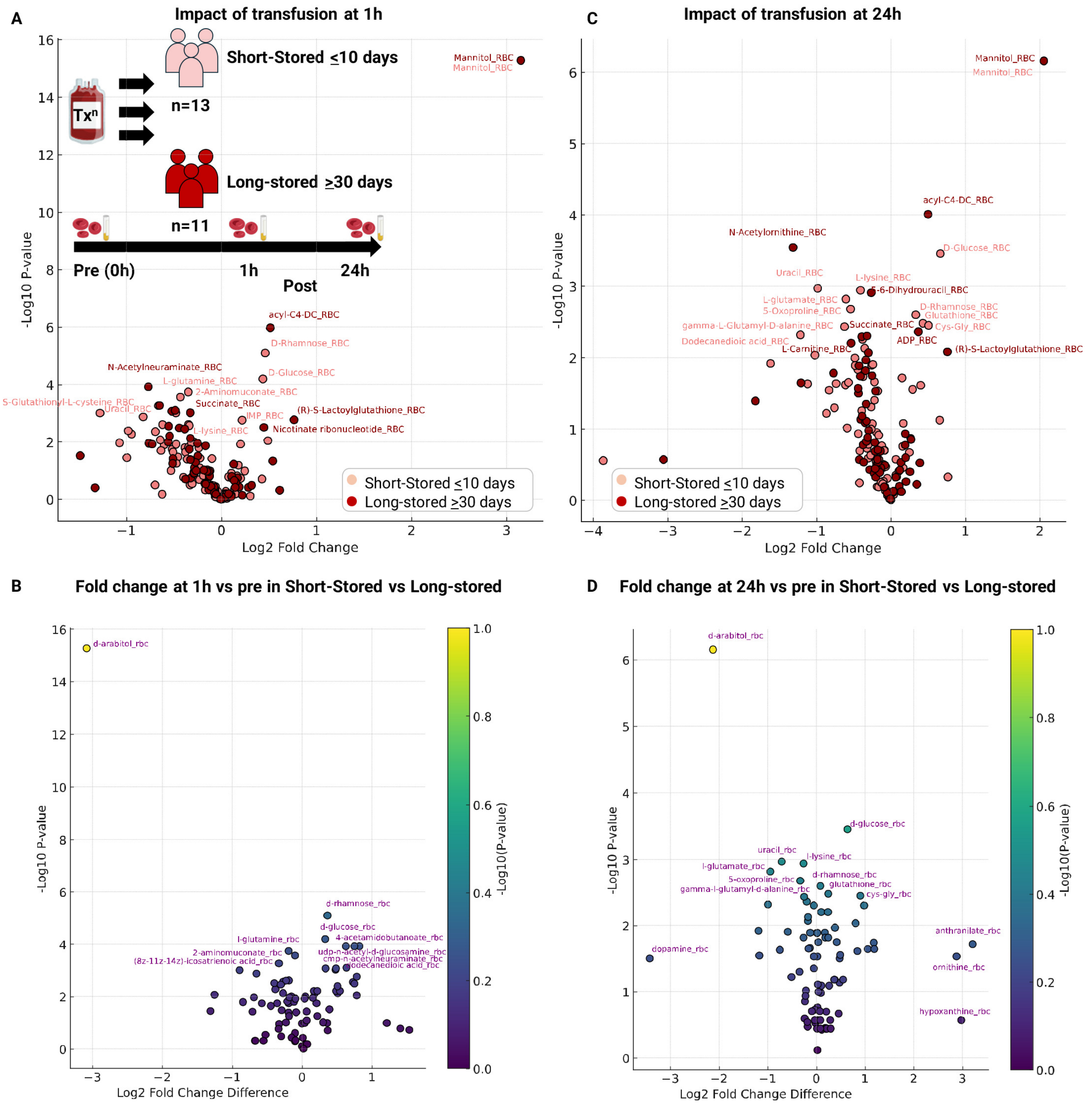

Supplementary Figure 1 – Metabolic changes at 1h (A-B) or 24h (C-D) vs pre-transfusion in plasma and RBCs of recipients with sickle cell disease. Results are either presented as volcano plots of pre- vs post-transfusion (**A-C**) or log2 fold change of post to pre-transfusion ratios (**B-D**).

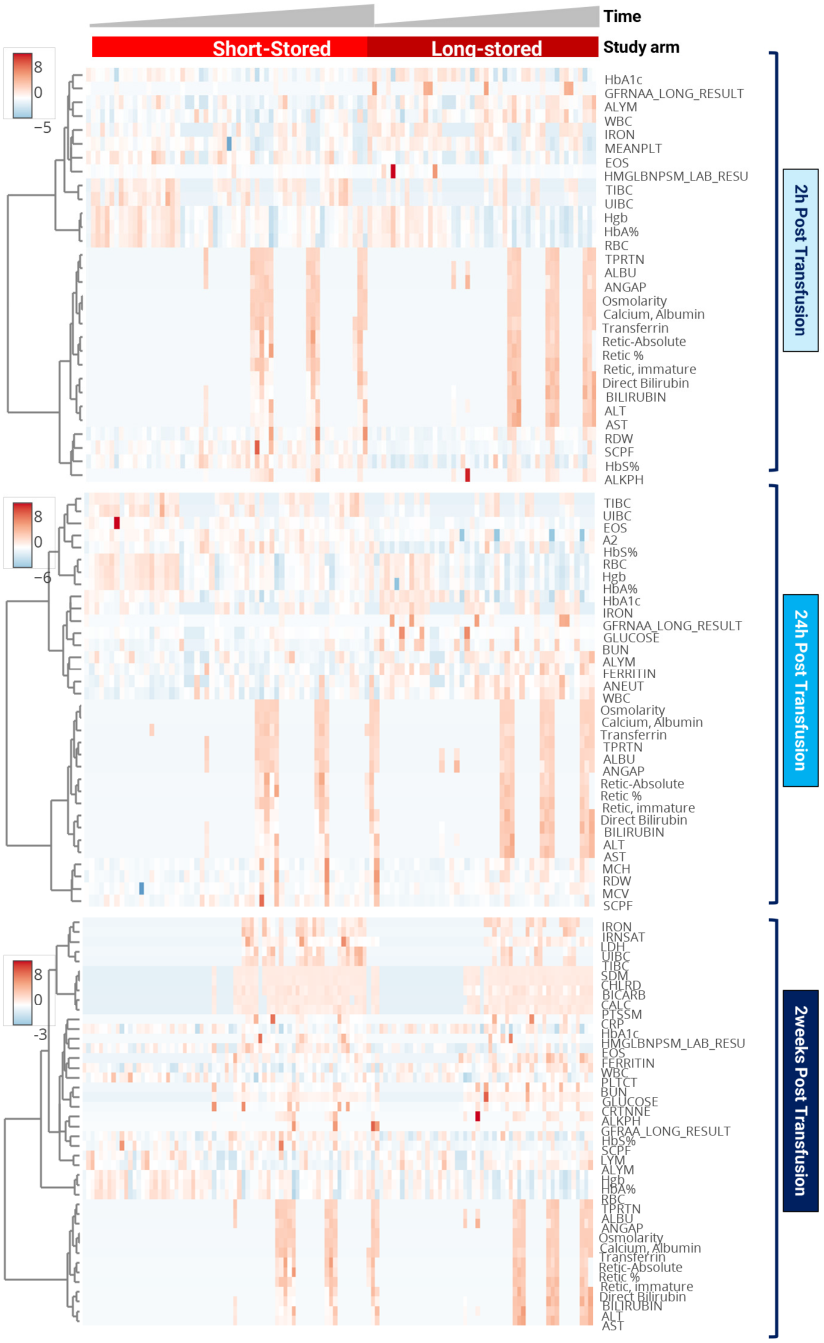

Supplementary Figure 2 – Heat map of the clinical impact of transfusion of pRBCs stored $\leq$ 10 days (fresh) or $\geq$30 days (long-stored) on the clinical chemistry and complete blood counts of recipients with sickle cell disease.

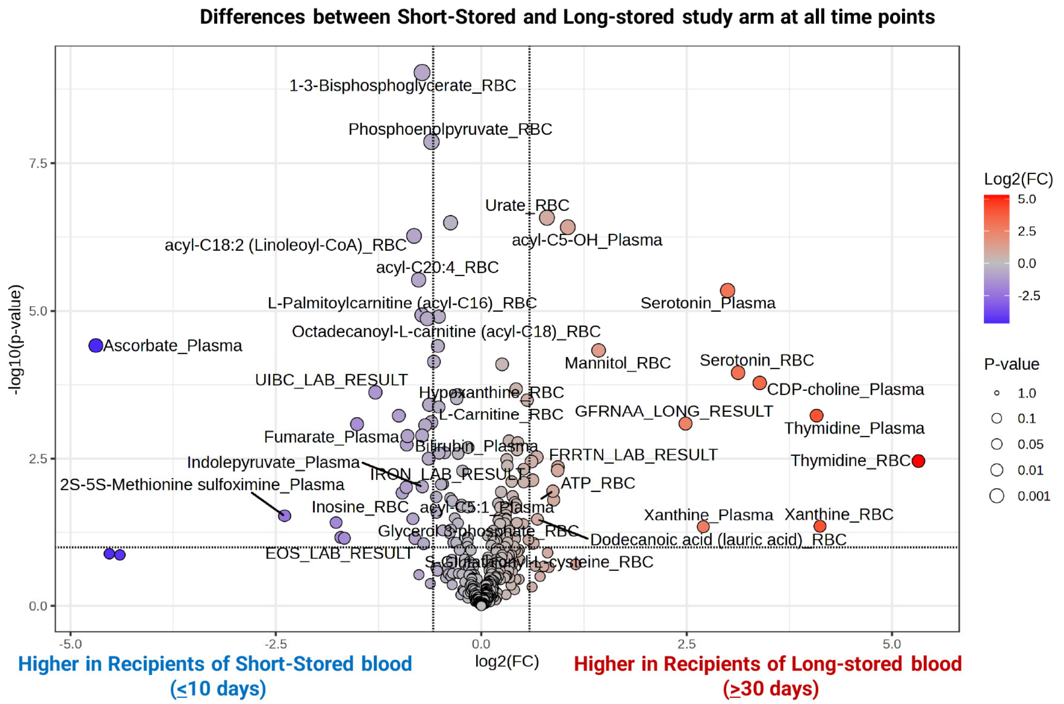

Supplementary Figure 3 – Metabolic and clinical differences in RBC and plasma of recipients of pRBCs stored ≤10 days or ≥30 days.

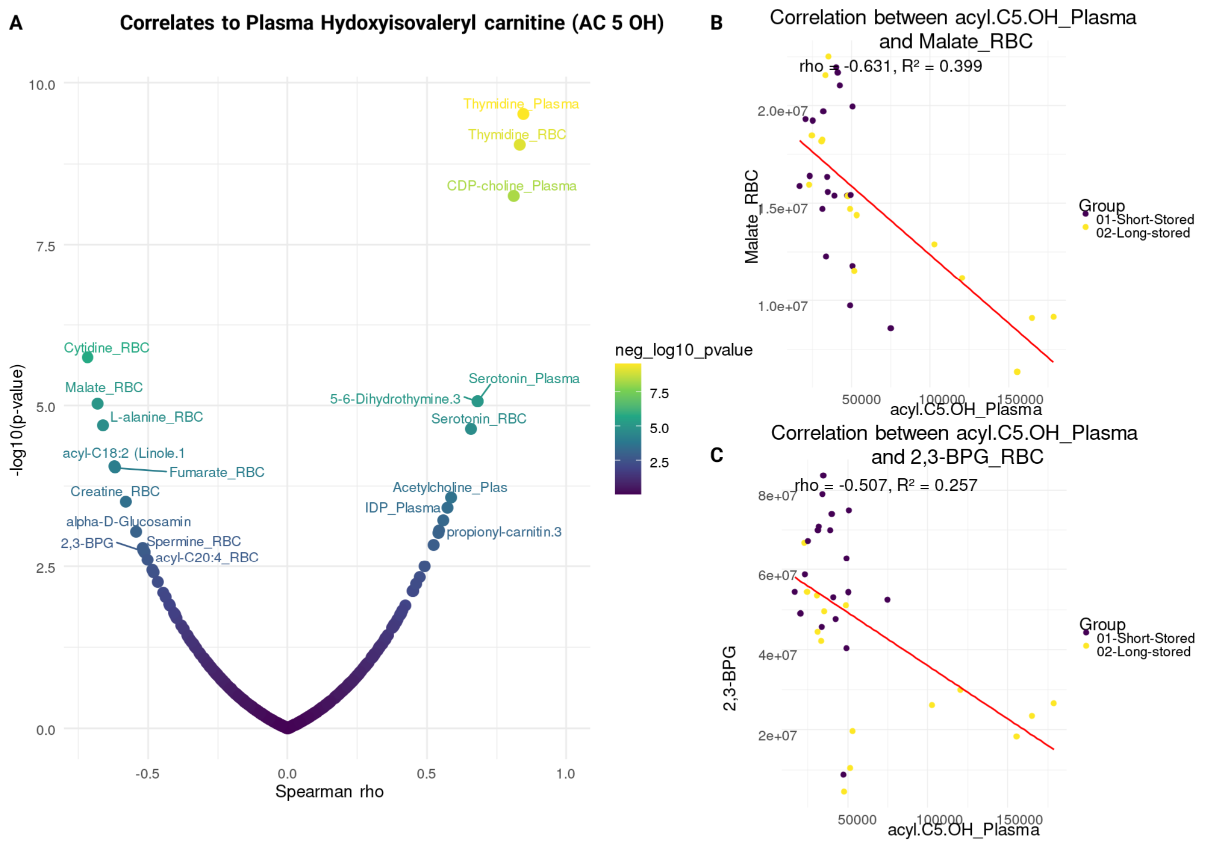

Supplementary Figure 4 – Example of the interactive ShinyApp for data analysis of the results presented in this study, with a focus on hydroxyisovaleryl-carnitine – a product of branched-chain amino acid metabolism.

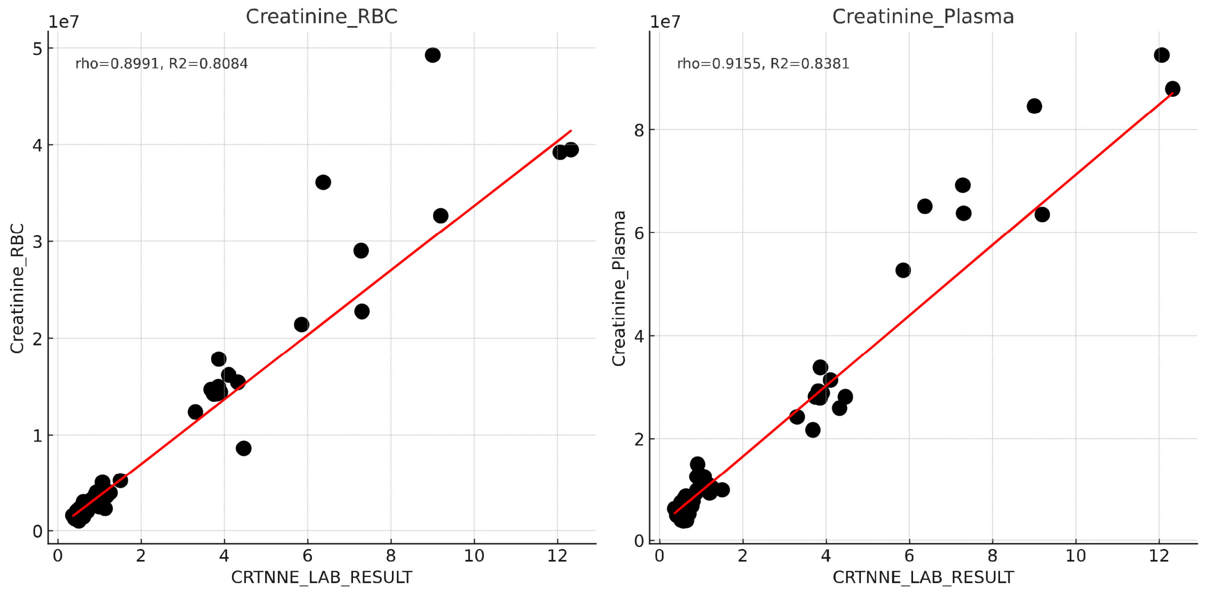

Supplementary Figure 5 – Correlation between RBC and plasma creatinine levels – as measured by mass spectrometry or CLIA-certified clinical chemistry assays.

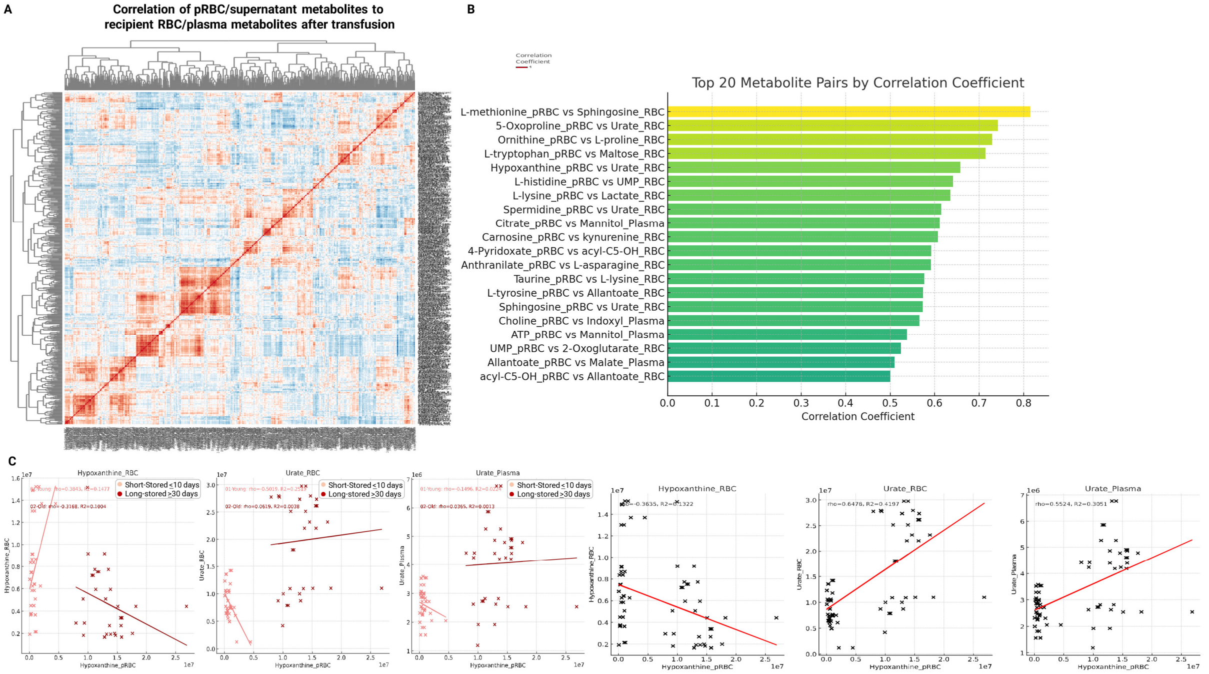

Supplementary Figure 6 – Correlation between metabolites in the blood bag that was transfused (pRBC or supernatants) and metabolites in the post-transfusion RBC and plasma in recipients with sickle cell disease. In **A-C**, results are shown as correlation matrix, bar plots of the top 20 strongest correlations, and related scatter plots, broken down by young vs old pRBC transfusion (light vs dark red, respectively).

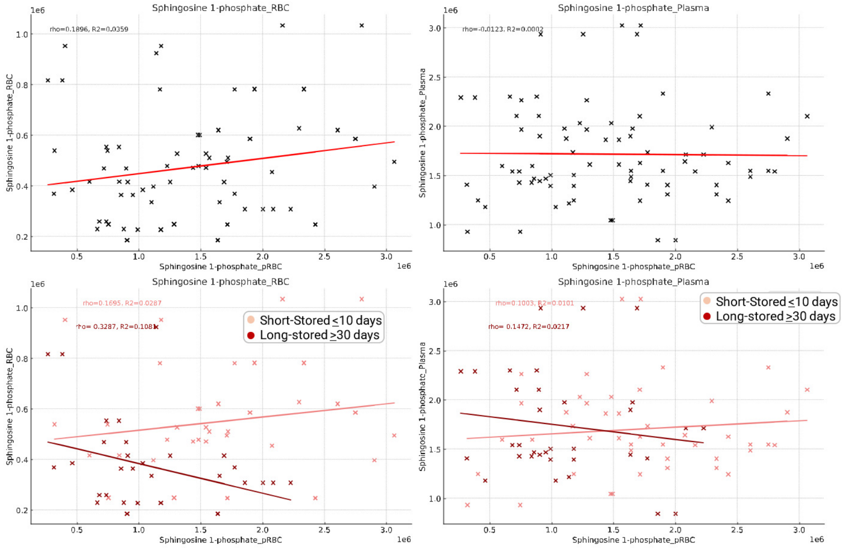

Supplementary Figure 7 – Levels of markers of hypoxia, like sphingosine 1-phosphate, poorly correlated between pRBC and recipients' RBC post-transfusion, or pRBC supernatants and recipients' plasma – independently of the storage age of transfused products.
